# A conformational checkpoint between DNA binding and cleavage by CRISPR-Cas9

**DOI:** 10.1126/sciadv.aao0027

**Published:** 2017-08-04

**Authors:** Yavuz S. Dagdas, Janice S. Chen, Samuel H. Sternberg, Jennifer A. Doudna, Ahmet Yildiz

**Affiliations:** 1 Biophysics Graduate Group, University of California, Berkeley, Berkeley, CA 94720, USA.; 2 Department of Molecular and Cell Biology, University of California, Berkeley, Berkeley, CA 94720, USA.; 3 Department of Chemistry, University of California, Berkeley, Berkeley, CA 94720, USA.; 4 Howard Hughes Medical Institute, University of California, Berkeley, Berkeley, CA 94720, USA.; 5 Physical Biosciences Division, Lawrence Berkeley National Laboratory, Berkeley, CA 94720, USA.; 6 Department of Physics, University of California, Berkeley, Berkeley, CA 94720, USA.

## Abstract

The Cas9 endonuclease is widely used for genome engineering applications by programming its single-guide RNA, and ongoing work is aimed at improving the accuracy and efficiency of DNA targeting. DNA cleavage of Cas9 is controlled by the conformational state of the HNH nuclease domain, but the mechanism that governs HNH activation at on-target DNA while reducing cleavage activity at off-target sites remains poorly understood. Using single-molecule Förster resonance energy transfer, we identified an intermediate state of *Streptococcus pyogenes* Cas9, representing a conformational checkpoint between DNA binding and cleavage. Upon DNA binding, the HNH domain transitions between multiple conformations before docking into its active state. HNH docking requires divalent cations, but not strand scission, and this docked conformation persists following DNA cleavage. Sequence mismatches between the DNA target and guide RNA prevent transitions from the checkpoint intermediate to the active conformation, providing selective avoidance of DNA cleavage at stably bound off-target sites.

## Teaser

A conformational checkpoint of Cas9 enables on-target cleavage while blocking access to the active state at off-target sites.

## INTRODUCTION

The RNA-guided endonuclease Cas9 is responsible for recognizing, unwinding, and cutting double-stranded DNA (dsDNA) targets as part of the type II CRISPR-Cas (clustered regularly interspaced short palindromic repeats–CRISPR-associated) adaptive immune system (*1*–*4*). DNA target recognition requires a short protospacer adjacent motif (PAM) sequence (5′-NGG-3′ for *Streptococcus pyogenes* Cas9) (*5*) and complementary base pairing with the 20-nucleotide (nt) targeting sequence of the guide RNA. Cleavage of the target strand (TS) and nontarget strand (NTS) is mediated by the conserved HNH and RuvC nuclease domains, respectively (*6*, *7*). By manipulating the sequence of the single-guide RNA (sgRNA), Cas9-sgRNA can be programmed to target any DNA sequence flanked by a PAM (*6*, *8*–*10*), making it a powerful genome-editing tool. However, promiscuous cleavage of off-target sites remains a major challenge (*5*, *11*–*14*). Efforts are in progress (*15*, *16*) to enhance the specificity of DNA targeting by Cas9 for potential therapeutic applications (*17*, *18*), which requires detailed mechanistic understanding of substrate-dependent activation of Cas9 for DNA cleavage.

**Fig. 1:**
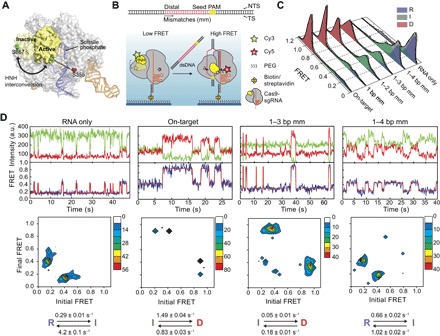
HNH conformational dynamics reveal a distinct I state as a function of PAM-distal mismatches. (**A**) Model shows HNH labeling sites under different conformations of Cas9, using sgRNA-bound (4ZT0) and dsDNA-bound (5F9R) structures. The cysteine-light Cas9 construct is labeled with Cy3 and Cy5 at S867C and S355C positions. (**B**) Top: Cas9 was incubated with 55-bp-long dsDNA substrates that include PAM and target sequences. Mismatches were introduced at the PAM-distal site. Bottom: DNA binding to Cas9 results in HNH interconversion, determined by a transition from a low to high FRET state. Scissors show the DNA cleavage sites. (**C**) Steady-state smFRET histograms for Cas9 in the absence and presence of 200 nM DNA targets. A multi-Gaussian fitting (black curve) reveals D, I, and R states of HNH. (**D**) Representative time traces (top), transition density plots (TDPs; middle), and rates of the major transitions in TDPs (bottom) for various DNA substrates. a.u., arbitrary units.

A previous bulk Förster resonance energy transfer (FRET) study revealed that the HNH conformation regulates DNA cleavage activity of both nuclease domains and is highly sensitive to RNA-DNA complementarity (*12*). After binding to a DNA target, *S. pyogenes* Cas9 undergoes large conformational rearrangements that enable the HNH active site to hydrolyze the TS scissile phosphate (Fig. 1A) (*19*). However, none of the published structures of Cas9 have captured the HNH domain at the cleavage site (*19*–*22*), and the molecular cues that govern transitions to the active conformation are not well understood. Because bulk methods are insensitive to underlying dynamics, whether the HNH domain only transiently switches to the active conformation or stably docks onto the active site for DNA cleavage remains unresolved. Furthermore, Cas9 cleaves only a subset of off-target sites to which it binds (*23*, *24*), but it remains unclear whether HNH conformational dynamics play a direct role in decoupling between off-target binding and cleavage. To address these questions, we monitored the conformational rearrangements of the HNH domain during DNA binding and cleavage using single-molecule FRET (smFRET).

## RESULTS

### HNH conformational dynamics reveal a conformational checkpoint before DNA cleavage

A cysteine-light *S. pyogenes* Cas9 variant was labeled at positions S355C and S867C with Cy3 and Cy5 dyes (Fig. 1, A and B), which remained fully functional for DNA binding and cleavage (fig. S1) (*12*). Cas9 was preassembled with the sgRNA and DNA substrate (Fig. 1B and table S1), surface-immobilized via the sgRNA, and imaged under total internal reflection excitation (Fig. 1C). Steady-state smFRET measurements of Cas9-sgRNA revealed distinct HNH conformations (fig. S2 and table S1). Without the DNA substrate, the majority of the complexes were in the RNA-bound (R) state [FRET efficiency (*E*_FRET_) = 0.19 ± 0.02, ± SEM]. Addition of 200 nM on-target DNA resulted in a near-complete loss of the R population and the appearance of the docked (D) population at 0.97 ± 0.01 *E*_FRET_. The D population was not observed with nontarget or no-PAM DNA (fig. S1), suggesting that this conformation requires stable RNA-DNA base pairing (*25*).

To understand how sensing of the RNA-DNA heteroduplex affects the HNH conformation (*19*), we introduced 1- to 4-bp (base pair) mismatches (mm) at the PAM-distal end of the target region (Fig. 1B). When Cas9 was premixed with these substrates, an intermediate (I) population emerged at 0.34 ± 0.03 *E*_FRET_ (Fig. 1C). As the number of mismatches was increased, we observed a steady decrease in the D population, coupled with an increase in I and R populations. Remarkably, the HNH domain was unable to attain its D conformation with a 1–4 bp mm DNA (Fig. 1C), consistent with the inability of Cas9 to cleave this substrate (*12*). We concluded that the D state represents the active conformation of the HNH domain for DNA cleavage. *E*_FRET_ values of the R, I, and D states are consistent with the distance between the labeled positions from available structures and the predicted cleavage-competent conformation of Cas9 (table S2) (*26*, *27*). Similar results were obtained with a reciprocal Cas9 variant (fig. S3) (*12*), verifying that our conclusions are not markedly affected by the dye labeling positions.

We analyzed individual smFRET trajectories to address whether HNH activation is completely prohibited or the residence time in the active conformation is reduced at off-target sites. In the absence of DNA, ~50% of Cas9-sgRNA complexes displayed a stable R conformation (fig. S4), whereas the remaining 50% briefly visits the I state (Fig. 1D and fig. S5), indicating that this conformation is not stable without DNA. When Cas9 was bound to its on-target (fig. S6), the majority (90%) of the complexes maintained a stable D conformation (fig. S4) and only 3% displayed dynamic transitions between the D and I states. In comparison to an on-target, a larger percentage (35%) of complexes assembled with a 1–3 bp mm DNA underwent transitions between the I and D states at a ~100-fold slower rate. The complexes bound to a 1–4 bp mm DNA transitioned only between the R and I states, whereas transitions to the D state were not observed (Fig. 1D). These results suggest that the I state serves as the conformational checkpoint of RNA-DNA complementarity before the HNH domain transitions to the D state.

### The HNH domain visits the checkpoint intermediate before accessing its active conformation

**Fig. 2:**
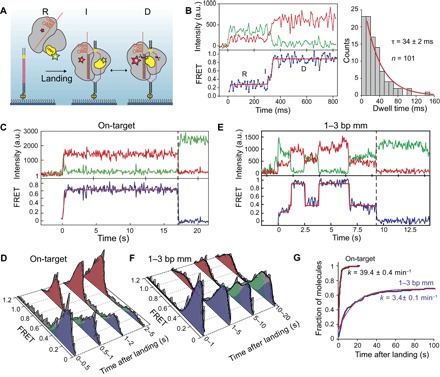
Real-time kinetics of HNH activation immediately after DNA binding. (**A**) Schematic for observation of Cas9 conformational dynamics upon landing onto surface-immobilized DNA. (**B**) Left: A representative smFRET trajectory recorded at 100 Hz shows a brief visit to the I state between initial on-target binding and transitioning into the D state. Right: Single exponential fit (red curve) to the I state dwell time histogram reveals its lifetime (τ, ±95% confidence interval). (**C** and **E**) A representative smFRET trajectory at 10 Hz of Cas9 after landing to an on-target and 1–3 bp mm DNA. *t* = 0 s and dashed vertical lines represent time of landing and acceptor photobleaching, respectively. (**D** and **F**) Time-dependent changes in the conformational distribution of Cas9 after DNA landing. (**G**) Cumulative distribution of first transition to the D state after DNA landing. Red curves show fit to a single exponential function (±95% confidence interval).

To test the checkpoint hypothesis, we determined the real-time kinetics of HNH activation immediately upon binding to surface-immobilized DNA (Fig. 2A). The complexes were in the R state when initially bound to DNA (Fig. 2B). Individual trajectories recorded at 100 Hz revealed that Cas9 visits the I state (τ = 34 ms) between initial landing and transitioning to the D state (Fig. 2B), consistent with the I state serving as the checkpoint between DNA binding and cleavage. The first transition to the D state occurs rapidly (39 min^−1^) after initial binding to an on-target DNA (Fig. 2, C, D, and G). Ninety-five percent of the complexes stably remained in the D state (Fig. 2C and fig. S7), and reversible transitions to the I and R states were rarely observed. With a 1–3 bp mm DNA, the first transition to the D state occurs at a ~10-fold slower rate relative to an on-target DNA (Fig. 2, E to G) and most of the complexes underwent reversible transitions after reaching the D state (Fig. 2D and fig. S7).

### HNH activation requires divalent cation but is independent of nuclease activity

**Fig. 3:**
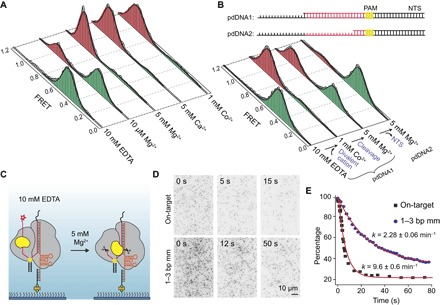
HNH activation requires divalent cation but is independent of nuclease activity. (**A**) smFRET histograms of Cas9 bound to on-target dsDNA in the absence and presence of a divalent cation. (**B**) Top: The on-target DNA was truncated at the 5′ end of the NTS one base after the target sequence (pdDNA1) and four bases after PAM (pdDNA2). Bottom: smFRET histograms of Cas9 bound to pdDNAs in the absence and presence of a divalent cation. (**C**) Cleavage of pdDNA1 was initiated by replacing EDTA with 5 mM Mg^2+^ and monitored by dissociation of the Cy5-labeled NTS 5′ end from Cas9. (**D**) Still images of Cy5-pdDNA1 bound to surface-immobilized Cas9 after Mg^2+^ addition (*t* = 0 s). (**E**) Percentage of Cy5 spots remain at the surface after Mg^2+^ flow. Red curves represent fit to single exponential decay (mean ± 95% confidence interval).

Next, we tested the roles of divalent cations and nuclease activity on conformational activation of the HNH domain. DNA cleavage activity of Cas9 requires Mg^2+^ at the catalytic site (*6*), yet it remains stably bound to the DNA target without Mg^2+^ (fig. S8). It was unclear whether Mg^2+^ enables transition to the D state or stabilizes the D state via interactions with the active site and DNA phosphate backbone (*19*). We observed that Cas9 is completely unable to transition to the D state without divalent cations (Fig. 3A). At low (10 μM) Mg^2+^, 29% of molecules were in the D conformation (Fig. 3A), and 50% of trajectories revealed I-to-D transitions (fig. S9). At 1 to 5 mM Mg^2+^, Ca^2+^, or Co^2+^, >85% of complexes populated the D state, demonstrating that divalent cations lower the threshold energy for the HNH domain to move to its active state. We observed the same divalent cation-dependent docking for catalytically dead dCas9 (fig. S10). Furthermore, Co^2+^ is unable to support DNA cleavage (fig. S8), but enabled transitions to the D state (Fig. 3A) (*6*), demonstrating that docking of the HNH domain is independent of nuclease activity.

To explore the stability of the D conformation after NTS release, we truncated to the 5′ end of the spacer in the NTS (pdDNA1) to prevent base pairing beyond the target region and enable dissociation of the NTS 5′ end from the complex after cleavage (Fig. 3B) (*28*). The complexes bound to pdDNA1 transitioned to the D state upon Co^2+^ addition. Unlike an on-target dsDNA, in which the NTS remains bound to the complex after cleavage, initiating the cleavage of pdDNA1 by Mg^2+^ addition reduced the stability of the D conformation. Furthermore, when Cas9 was bound to a distinct substrate that lacks the entire 5′ end of NTS upstream of the cleavage site (pdDNA2), most of the complexes remained in the I state in Mg^2+^. Because the HNH domain reverts back to the I state upon NTS release and cannot dock in the absence of the NTS, the 5′ end of the NTS is necessary for stabilizing the D state (*29*).

To determine whether stable docking of HNH is rate-limiting for the DNA cleavage activity, we designed a single-molecule stopped flow assay to monitor DNA cleavage activity of individual Cas9 complexes in real time. In this assay, pdDNA1 was labeled with Cy5 at the 5′ end of NTS and preassembled with Cas9 in 10 mM EDTA, and then complexes were immobilized via the sgRNA (Fig. 3C). Replacement of EDTA with 5 mM Mg^2+^ resulted in disappearance of 80% of the Cy5 spots at a rate of 0.16 s^−1^ (Fig. 3D and movie S1), which represents DNA cleavage and NTS release. Because docking of the HNH domain occurs about fourfold faster than DNA cleavage, it is not rate-limiting for cleavage of the on-target DNA. However, when Cas9 was preassembled with a 1–3 bp mm DNA, HNH docking and DNA cleavage (Figs. 2G and 3E and movie S2) occurred at comparable rates, suggesting that HNH docking is a rate-limiting step for the cleavage of this substrate.

### Truncation of the guide RNA traps the HNH domain in the checkpoint intermediate with fewer mismatches on the DNA

**Fig. 4:**
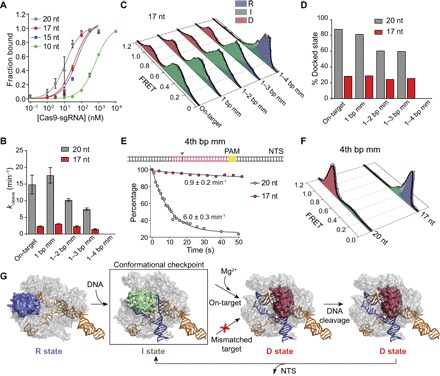
Truncation of the gRNA traps the HNH domain in the checkpoint intermediate with fewer mismatches on the DNA. (**A**) On-target DNA binding assay with 20-nt and truncated gRNAs. (**B**) Bulk cleavage rates of the DNA substrates by Cas9 assembled with 20- and 17-nt gRNAs. (**C**) Steady-state smFRET histograms of Cas9 guided with a 17-nt gRNA. (**D**) D population of Cas9 guided with 20- and 17-nt gRNAs. (**E**) Top: A single mismatch was introduced at the 4th bp after the PAM-distal end (blue arrowhead). Bottom: Real-time cleavage of 4th bp mm by Cas9 guided with 20- and 17-nt gRNAs. Black curves represent fit to a single exponential decay (mean ± 95% confidence interval). (**F**) Steady-state smFRET histograms of Cas9 with 20- and 17-nt gRNAs bound to 4th bp mm. (**G**) Model for substrate-dependent HNH activation. DNA binding triggers transition from R (blue) to I (green) conformation, which serves as a conformational checkpoint between DNA binding and cleavage. In Mg^2+^, recognition of an on-target locks HNH in the catalytically active D conformation (red), which is destabilized after NTS release. HNH activation is prohibited when the RNA-DNA complementarity drops below a threshold (red cross).

Truncating the 5′ end of the guide RNA (gRNA) has been shown to reduce off-target effects with Cas9 (*30*). It has been proposed that truncation of the gRNA leads to a reduction in DNA affinity, in a way that Cas9 can still bind to an on-target site, but the affinity is too low to allow binding to off-target sites (*30*). Cas9 with a 17-nt gRNA has fourfold lower on-target affinity (Fig. 4A) and reduced cleavage rate (Fig. 4B) compared to a standard (20-nt) gRNA. Further truncation of the gRNA reduced the DNA binding affinity (Fig. 4A) and fully abolished on-target cleavage (fig. S1). To test whether the reduction in cleavage activity is due to the inability of Cas9 to bind the DNA substrate, we measured HNH docking and the cleavage activity of DNA-bound Cas9 complexes guided with a 17-nt gRNA at a single molecule level. When Cas9 guided with a 17-nt gRNA was bound to its on-target DNA substrates (on-target, 1 bp mm, 1–2 bp mm, and 1–3 bp mm), we observed a lower occupancy (~20%) of the D state, whereas most of the complexes remained in the I state (Fig. 4, C and D), which manifests with the reduced cleavage rate (Fig. 4B). Binding to a 1–4 bp mm DNA, which introduces a single mismatch relative to the 17-nt sgRNA, completely prohibits cleavage activity and transitions to the D state (Fig. 4, B and D). When a single mismatch was introduced at the 4th bp from the PAM-distal end (4th bp mm), Cas9 guided with a 17-nt gRNA was unable to dock and cleave the DNA, whereas a 20-nt gRNA enabled transitions to the D state and cleaved the DNA at near on-target rates (Fig. 4, E and F). Therefore, truncation of gRNA increases cleavage specificity by prohibiting HNH activation with a single mismatch at the PAM-distal end, not by the reduction of off-target binding.

## DISCUSSION

Here, we detected conformational activation of Cas9 for DNA cleavage (Fig. 4G). Upon binding to an on-target DNA, the HNH domain transitions from the RNA-bound to catalytically active D conformation. Binding to a divalent cation is a prerequisite for HNH activation. HNH remains docked to the catalytic site after cleavage (*5*, *13*, *14*, *28*), and this conformation is destabilized upon NTS release. These results explain why the active conformation of the HNH domain was not observed without the full dsDNA substrate or divalent cations (*19*–*22*). We speculate that the cleavage-competent conformation may be captured with wild-type (WT) Cas9 in Co^2+^ or with dCas9 in Mg^2+^.

In the presence of mismatched targets, the emergence of the catalytically inactive I state highlights a conformational checkpoint through which the HNH nuclease must pass to occupy the active conformation and achieve DNA cleavage (Fig. 4G). The energetic barrier regulating HNH activation is sensitive to mutations on the DNA substrate. Docking into the active state is blocked when complementarity drops below a threshold, and Cas9 remains in the checkpoint intermediate. These results demonstrate the inherent conformational specificity of Cas9 and provide a structural and kinetic explanation for the decoupling of DNA binding and cleavage.

Truncation of the sgRNA to 17 nt increases the cleavage specificity of Cas9 by preventing HNH activation with a single mutation at the PAM-distal end. This is achieved at the expense of reduced on-target cleavage, because the HNH domain mostly remains in the checkpoint intermediate without sufficient RNA-DNA base pairing. For future genome engineering applications, the smFRET approach developed here can be coupled with targeted mutagenesis to design and test high-fidelity Cas9-sgRNA complexes with minimal off-target cleavage while maintaining optimal on-target cleavage activity.

## MATERIALS AND METHODS

### Protein purification and labeling

WT Cas9, dCas9 (D10A/H840A), Cas9_HNH-1_ (C80S/S355C/C574S/S867C), dCas9_HNH-1_ (D10A/C80S/S355C/C574S/H840A/S867C), and Cas9_HNH-2_ (C80S/C574S/S867C/N1054C) were purified as described (*12*). Dye-labeled Cas9 was prepared as previously described (*12*), with the following modifications: Labeling reactions contained 10 μM Cas9, 200 μM Cy3-maleimide, and 400 μM Cy5-maleimide (GE Healthcare). For steady-state measurements, regular cyanine dyes (GE Healthcare) were used. Free Cy3- and Cy5-maleimeide dyes were separated from labeled Cas9 through Micro Bio-Spin 6 columns (Bio-Rad) that were buffer-exchanged into Cas9 gel filtration buffer [20 mM tris-HCl (pH 7.5), 200 mM KCl, 5% glycerol, and 1 mM tris(2-carboxyethyl)phosphine (TCEP)]. To enhance the photostability in dynamic FRET measurements, Cas9 was labeled with Cy3 and Cy5 derivatives, LD550-MAL and LD650-MAL (Lumidyne Technologies). Free dyes were separated from labeled Cas9 by gel filtration on a Superdex 200 Increase 10/300 GL column [20 mM tris-HCl (pH 7.5), 200 mM KCl, 5% glycerol, and 1 mM TCEP].

### Nucleic acid preparation

sgRNA templates were polymerase chain reaction–amplified and cloned into Eco RI and Bam HI sites in pUC19 (table S1), transcribed, and purified in vitro, as previously described (*12*). DNA oligonucleotides and 5′ biotinylated NTSs (table S1) were ordered synthetically (Integrated DNA Technologies), and DNA duplexes were prepared and purified by native polyacrylamide gel electrophoresis (PAGE), as described.

### DNA bulk cleavage and binding assays

DNA duplex substrates were 5′-[^32^P]–radiolabeled on both strands. For cleavage experiments, Cas9 and sgRNA were preincubated at room temperature for 10 min in 1× binding buffer [20 mM tris-HCl (pH 7.5), 100 mM KCl, 5 mM MgCl_2_, 1 mM dithiothreitol (DTT), 5% glycerol, and heparin (50 μg/ml)] before initiating the reaction by addition of DNA duplexes. DNA cleavage experiments were performed and analyzed as previously described (*12*).

Binding assays were performed as previously described (*12*), with the following modifications: For the 0 mM MgCl_2_ condition, binding reactions were conducted in 1× binding buffer + 1 mM EDTA in the absence of MgCl_2_ [20 mM tris-HCl (pH 7.5), 100 mM KCl, 1 mM DTT, 1 mM EDTA, 5% glycerol, and heparin (50 μg/ml)] and resolved on 8% native PAGE [0.5× tris-borate EDTA (TBE) + 1 mM EDTA, without MgCl_2_] at 4°C. For the 5 mM MgCl_2_ condition, binding reactions were conducted in 1× binding buffer and resolved on 8% native PAGE (0.5× TBE + 5 mM MgCl_2_) at 4°C. Experiments were replicated at least three times, and presented gels are representative results.

### Sample preparation for smFRET assay

Quartz slides coated with 99% polyethylene glycol (PEG) and 1% biotinylated PEG were acquired from MicroSurfaces Inc. Air-tight sample chamber was prepared by sandwiching double-sided tape between quartz slides and coverslips. To prepare the slides for molecule deposition, the PEG surface was preblocked with casein (10 mg/ml) incubated for 10 min. Flow chamber was washed with 1× binding buffer and then incubated with 20 μl of streptavidin (1 mg/ml) for 10 min. Excess streptavidin was washed away with 40 μl of 1× binding buffer. All the smFRET experiments were performed using Cas9_HNH-1_, with the exception that data shown in figs. S3 and S10 were collected using Cas9_HNH-2_ and dCas9_HNH-1_ constructs, respectively.

Two separate experimental geometries were used for smFRET experiments. To surface immobilize Cas9 from its sgRNA, 50 nM sgRNA was hybridized to a 5′ biotin-DNA tether. Cas9-sgRNA complexes were preassembled by mixing 50 nM Cas9, 50 nM sgRNA-biotin, and 200 nM DNA substrate in 1× binding buffer and incubating for 10 min. The sample was spun at 16,000*g* at 4°C for 5 min. The supernatant was diluted to 100 pM, flown into the sample chamber, and incubated for 10 min. To surface immobilize Cas9 from its DNA substrate, 1 nM biotinylated DNA substrate [NTS (5′ biotin) hybridized to TS] was flown into the sample chamber and incubated for 10 min. The chamber was washed with 1× binding buffer. Cas9 (50 nM) and sgRNA (50 nM) were mixed in 1× binding buffer and incubated for 10 min. The sample was spun at 16,000*g* at 4°C for 5 min. The supernatant was diluted to 100 pM, flown into the sample chamber, and incubated for 10 min. Before data acquisition, the sample chamber was washed with 1× binding buffer and 20 μl of imaging buffer [glucose oxidase (1 mg/ml), catalase (0.04 mg/ml), 0.8% dextrose, and 2 mM Trolox in 1× binding buffer].

To perform smFRET measurements of Cas9-sgRNA complexes that landed onto a DNA-immobilized surface, 400 pM Cas9-sgRNA mixture was flown into the sample chamber in 100 μl of imaging buffer during data acquisition. After recording each movie, the imaged area was photobleached with an intense laser beam, and fresh Cas9-sgRNA mixture in imaging buffer was flown into the chamber while imaging the bleached region. This approach prevented crowding of the imaged area with fluorescent spots and enabled more reliable detection of the molecules that landed onto the surface during data acquisition.

### Microscopy and data acquisition

A prism-type total internal reflection fluorescence microscope was set up using a Nikon Ti-E Eclipse inverted fluorescence microscope equipped with a 60× 1.20 N.A. Plan Apo water objective and the Perfect Focus System (Nikon). A 532-nm solid-state laser (Coherent Compass) and a 633-nm HeNe laser (JDSU) were used for Cy3 and Cy5 excitation, respectively. Cy3 and Cy5 signals were split into two channels using an OptoSplit II image splitter (Cairn Instruments) and imaged separately on the same electron-multiplied charged-coupled device camera (512 × 512 pixels, Andor iXon EM^+^). Effective pixel size of the camera was set to 267 nm after magnification. Movies for steady-state FRET measurements were acquired at 10 Hz under 0.3 kW cm^−2^ 532-nm excitation. For DNA landing assays, smFRET data were acquired at 100, 10, and 2 Hz under 1.0, 0.3, and 0.05 kW cm^−2^ 532-nm excitation, respectively.

### Observation of real-time DNA cleavage

The on-target (pdDNA1-Cy5) and 1–3 bp mm [pdDNA1 (1- to 3-bp mm)–Cy5] DNA substrates were truncated at the 5′ end of NTS, missing the flanking sequence at the PAM-distal site, and labeled with Cy5. Cy3-Cas9 (50 nM), 50 nM sgRNA-biotin, and 200 nM DNA were mixed in precleavage buffer [20 mM tris-HCl (pH 7.5), 100 mM KCl, 10 mM EDTA, 1 mM DTT, 5% glycerol, and heparin (50 μg/ml)] and incubated for 10 min. The sample was spun at 16,000*g* at 4°C for 5 min. The supernatant was diluted to 100 pM, flown into the sample chamber, and incubated for 10 min. Before data acquisition, the sample chamber was washed with precleavage buffer, and 20 μl of imaging buffer was prepared in precleavage buffer. Movies were acquired at 10 Hz under 0.05 kW cm^−2^ excitation intensity of the 633-nm beam. To initiate cleavage, flow chamber was washed with imaging buffer prepared in 1× binding buffer (containing 5 mM Mg^2+^) 5 s after the start of image acquisition. The number of molecules on the surface at the beginning of a movie was normalized to 100%. The percent of Cy5 spots that remained on the surface after Mg^2+^ addition was fitted to a single exponential decay to calculate the cleavage rate. Cy5 photobleaching was corrected by performing the assay under the same imaging conditions without initiating the DNA cleavage.

### Data analysis

The two Cy3 and Cy5 channels were registered with each other using fiducial markers (20-nm-diameter Nile Red Beads, Life Technologies) before each data acquisition. Colocalized Cy3 and Cy5 spots were analyzed for the FRET signal. Individual Cy3/Cy5 pairs that were photobleached in one step and that showed anti-correlated changes in fluorescence intensity were included in histograms. Donor-only molecules were excluded from the analysis, and FRET values were corrected for donor leakage into the acceptor channel. This procedure excluded the molecules labeled with a single dye or with two dyes of the same type. FRET histograms were constructed as reported previously (*31*). Each smFRET trace contributed equally to the steady-state FRET histograms. The histograms were normalized to determine the percentage of distinct FRET populations.

For dynamic FRET analysis, we selected the traces that showed at least one dynamic FRET transition within the time frame of movie acquisition (50 to 100 s) or before Cy3 or Cy5 photobleaching. The average lifetime of smFRET trajectories was approximately 50, 10, and 2 s for movies recorded under 0.05, 0.3, and 1.0 kW cm^−2^ excitation intensity, respectively. Raw smFRET trajectories were fitted to a step function using a maximum evidence algorithm in vbFRET. The maximum number of distinct FRET states was set to either two or five, and 50 fitting attempts were made for each trace. TDPs were plotted using a custom code written in MATLAB. The reverse cumulative histogram of each transition in a TDP was plotted, and transition rates of major transitions were calculated by fitting these histograms to a single exponential decay. To track the initial HNH conformational dynamics following DNA binding, the molecules that landed on the surface after the acquisition of the first frame of the movie were determined using smCamera.

## SUPPLEMENTARY MATERIALS

Supplementary material for this article is available at http://advances.sciencemag.org/cgi/content/full/3/8/eaao0027/DC1 fig. S1. Steady-state smFRET measurements and bulk cleavage assays of Cas9 labeled with Cy3/Cy5 FRET pairs. fig. S2. Representative smFRET trajectories reveal three different stable conformations of Cas9 under saturating DNA concentrations. fig. S3. Steady-state smFRET histograms of a reciprocal Cas9 variant. fig. S4. DNA immobilization of Cas9-sgRNA to the PEG surface eliminates the complexes that are unable to bind the DNA. fig. S5. Dwell time analysis of dynamic transitions of Cas9 in the absence and presence of the DNA substrates. fig. S6. Specific binding of Cas9 to the on-target DNA substrate immobilized to the PEG surface. fig. S7. Conformational dynamics of the HNH domain observed at 2 Hz. fig. S8. DNA cleavage activity of Cas9 in the presence of various divalent cations. fig. S9. Conformational dynamics of the HNH domain in the presence of 10 μM Mg^2+^. fig. S10. smFRET histograms of dCas9 bound to on-target dsDNA in the absence and presence of a divalent cation. table S1. List of RNA and DNA substrates used in this study. table S2. Expected and measured *E*_FRET_ values for Cas9_HNH-1_ and Cas9_HNH-2_. movie S1. Real-time cleavage of an on-target pdDNA1 by Cas9. movie S2. Real-time cleavage of a 1–3 bp mm pdDNA1 by Cas9. Reference (*32*)
